# Prevalence of primary headaches: it is not the behavior, but still we have to pay attention to it!

**DOI:** 10.1007/s10194-011-0319-3

**Published:** 2011-02-23

**Authors:** Julio Pascual

**Affiliations:** Neuroscience Area, Service of Neurology, University Hospital “Central de Asturias”, Calle Celestino Villamil s/n, 33006 Oviedo, Spain

Tension-type headache and migraine rank among the most common and disabling disorders worldwide. Headache burden has both individual—decreasing the quality of life of sufferers—and socio-economic—with absenteeism and higher health care consumption—consequences. This negative impact is especially impressive in the middle-aged women, where the prevalence of chronic migraine is above 5% of the general population [1], which leads to daily or almost daily analgesic overuse in around one-third of the cases [2]. The management of these primary headaches, and especially of those with frequent or chronic migraine attacks, therefore, must be taken seriously.

Could we decrease headache prevalence by eliminating some negative or unhealthy lifestyle habits? There is no doubt that, especially for migraine, factors such as sedentarism [3–5], obesity [6, 7], smoking [5, 8] or alcohol [5, 9] consumption are attack precipitants, what can be responsible for an impairment or even chronification. The role of these, and other, lifestyle habits as potential etiological factors leading to or influencing migraine or tension-type headache is not so clear. Reported data in this regard are first contradictory and second difficult to interpret, as these lifestyle factors can be both causes and effects for primary headaches. For instance, both tobacco and alcohol consumption have been associated with an increase or decrease in headache prevalence [5, 8–10]. A plausible explanation for these contradictory results could be that both alcohol and tobacco are in fact precipitants—which justifies those studies showing an increase in prevalence in drinkers or smokers—, but that subjects with headache disorders may be abstaining from alcohol or tobacco—this explaining studies showing an increase in prevalence in abstainers. It is also important to notice that some of the associations observed between lifestyle habits and primary headaches can be mediated by confounding factors. For instance, the statistically significant univariate association between smoking for obesity and headache prevalence disappears when adjusted for socio-economic factors [11].

In this issue of JHP, Winter and colleagues [12] again analyse a potential relationship between the prevalence of primary headaches and four common lifestyle factors: alcohol consumption, smoking, physical activity, and overweight. Their analysis is a step forward as they obtain face-to-face data from more than 7,000 subjects participating in three different epidemiological surveys carried out in Germany. Even taking into account their limitations, such as the cross-sectional (not prospective) nature of the data, the relevant variations in the distribution of lifestyle factors between the three regions, or the rather high age (35–75 years old) of the interviewed subjects, their results are straightforward: there was no consistent association between tension-type headache or migraine prevalence and any of these lifestyle factors. Therefore, the first message to our patients is that they are not experiencing headaches due to an “unhealthy” behavior in their habits, which should not be considered as etiological factors for experiencing headaches. This does not mean that a healthy behavior of lifestyle factors does not contribute to a much better management and quality of life in our migraine or tension-type headache patients. The German paper does not address this crucial point, but in general a critical analysis of current scientific literature convincingly shows an association between both following a negative lifestyle behavior and experiencing a higher number of attacks [3–11] with a consequently higher risk of suffering from ischemic events [13]. Contrary to heredity and genes, lifestyle habits do not seem to play a role in the etiology of primary headaches, but they should not be forgotten within the necessary global management approach of our headache patients.
